# Saturation and beating of acoustic phonon oscillations excited near the exciton resonance of strained polar ZnO/Zn_0.8_Mg_0.2_O multiple quantum wells

**DOI:** 10.1039/c7ra11702g

**Published:** 2018-02-20

**Authors:** Wei-Rein Liu, Ja-Hon Lin, Jyun-Sian Chen, Hsin-Ming Cheng, Sheng-Jie Li, Hou-Ren Chen, Chia-Hung Hsu, Wen-Feng Hsieh

**Affiliations:** National Synchrotron Radiation Research Center Hsinchu 30076 Taiwan liu.weirein@nsrrc.org.tw; Department of Electro-Optical Engineering, National Taipei University of Technology Taipei 10608 Taiwan jhlin@ntut.edu.tw; Material and Chemical Research Laboratories, Industrial Technology Research Institute Hsinchu 31040 Taiwan; Department of Photonics, Institute of Electro-Optical Engineering, National Chiao Tung University Hsinchu 30010 Taiwan

## Abstract

Saturation and beating of coherent acoustic phonon (CAP) oscillations were observed and attributed to the screening of a built-in electric field with increasing pump power using degenerate pump–probe measurements near the exciton resonance of polar ZnO/Zn_0.8_Mg_0.2_O multiple quantum wells (MQWs). After purifying the CAP signals by using an empirical mode decomposition, we found not only that the CAP amplitude follows the trend of the band gap renormalization (BGR) and shows saturation at high pump power, but also that the CAP oscillation period coincides with that of the MQWs, consistent with the XRD and TEM results. An additional low-frequency oscillation modifying the CAP signal is revealed due to the negative change in refractive index caused by BGR as the pump power increases.

## Abstract 2

Coherent acoustic phonons and carrier dynamics in ZnO/Zn_0.8_Mg_0.2_O multiple quantum wells from the perturbation of the built-in electric field are investigated using a degenerate pump–probe measurement technique near the exciton resonance.

## Graphical abstract

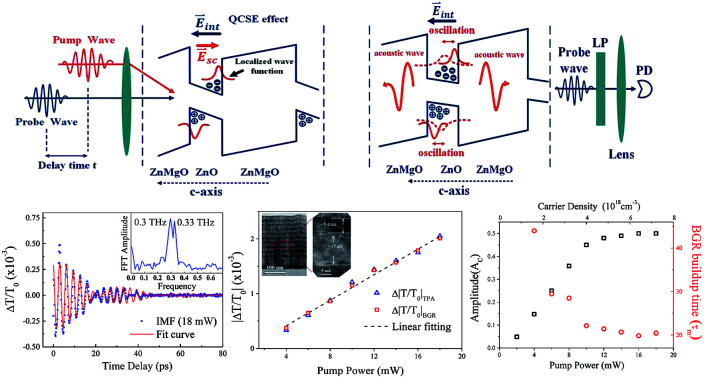

## Introduction

Over the past few decades, coherent phonon oscillations with frequencies in the terahertz range have attracted considerable attention because of their practical applications in phonon spectroscopy, nano-ultrasonic imaging^1,2^ and ultrafast modulation of optics and electronics. The mechanisms which generate coherent phonon oscillation have been studied extensively. Several mechanisms have been proposed to explain those oscillations, including impulsive stimulated Raman scattering (ISRS) for transparent media^4,5^ and the most frequently cited displacive excitation of coherent phonons (DECP) mechanism or recently resonant ISRS in opaque materials.^3^ The generation of photo-induced coherent acoustic phonon (CAP) oscillations is strongly correlated with electron–phonon and photon–phonon coupling mechanisms,^6^ which provide new physical insights into the properties of acoustic phonons and their interactions with surrounding carriers in complex and correlated systems.

By changing the period width of multiple-quantum wells (MQWs) providing efficient confinement of carriers or superlattices (SLs), the CAP oscillation frequency can be tuned. The oscillation frequency *ν* is related to the SL period *Λ*_SL_ and the sound velocity *c*_s_ in the medium by the formula: *ν* = *c*_s_/*Λ*_SL_.^7^ Due to the artificial periodicity the folding of the acoustic branches induced additional optical phonon-like modes in the artificial lattice, termed as zone folded longitudinal acoustic phonons, with non-zero frequencies at the Brillouin zone center (*q* ≅ 0). This enables the observation of higher frequency zone-folded CAP oscillations. Different from the CAPs generated in a GaAs material system, originating from the deformation potential coupling,^8–10^ large amplitude CAP oscillations in InGaN MQWs were observed due to the electron–phonon interaction being enhanced by the strong piezoelectric coupling mechanism.^11,12^

As a counterpart to GaN, because of the stronger excitonic oscillator strength and the larger exciton binding energy (∼60 meV) at room temperature (RT), wide-band-gap ZnO semiconductors have become promising candidates for various UV photonic devices, *e.g.*, LEDs^13^ and RT polariton lasers.^14^ Due to their strong piezoelectricity, they can also be used as sensors and transducers. Recently, the ultrafast carrier dynamics of polar^15,16^ and non-polar^17,18^ ZnO epitaxial films have been investigated through optical pump–probe transient differential reflection (TDR) spectroscopy. Furthermore, the generation of CAPs in a 600 nm *c*-ZnO film on silicon through Brillouin scattering had also been demonstrated when the excited photon energy was set below the exciton resonance of the sample.^19^

ZnO/ZnMgO MQW structures enhance the localization of carriers and photons in the well regions and so increase the exciton binding energy and interaction strength, nevertheless, being grown along the *c*-axis leads to an internal electric field being exerted across the quantum well which causes a quantum-confined Stark effect (QCSE).^20^ This internal electric field is attributed to spontaneous and piezoelectric polarizations, resulting from lattice mismatches between the well and barrier materials. However, to the best of our knowledge, an investigation into the correlation of the THz CAP oscillations with the carrier dynamics in strained polar ZnO/ZnMgO MQWs has never been reported.

In this study, the structural properties of ten-period ZnO/Zn_0.8_Mg_0.2_O MQWs (ZnO/ZnMgO MQWs) on (0001) sapphire substrates grown using pulsed laser deposition (PLD) were thoroughly examined using X-ray diffraction (XRD) and transmission electron microscopy (TEM). By performing degenerate pump–probe transient differential transmission (TDT) and reflection (TDR) spectroscopy, the sub-THz CAP and carrier dynamics in *c*-axis ZnO/ZnMgO MQWs were investigated using a photo-excitation energy set between the band edge and the exciton resonance of the ZnO well layers. The mechanism of CAP generation is attributed to the internal electric field being perturbed by the photocarrier screening field. The CAP signal was analyzed by using the empirical mode decomposition,^21,22^ and the noise and the low-frequency oscillation signal can be filtered out. The results indicate that the CAP oscillation is strongly correlated with band gap renormalization (BGR).

## Experimental section

### Sample preparation

Ten-period ZnO/ZnMgO MQWs were grown on double-side-polished (0001) sapphire substrates using PLD. A KrF excimer laser with a wavelength of 248 nm was focused on commercial hot-pressed stoichiometric ZnO (4N), Zn_0.97_Mg_0.03_O (3N) and Zn_0.8_Mg_0.2_O (3N) targets with a pulse energy density of ∼2–3 J cm^−2^. To minimize the effect of magnesium diffusion into the well layers by the thermal driving force at high growth temperatures, we adopted a temperature-gradient control method, in which the growth temperature of the barriers was set lower than that of the well layers. Prior to fabrication of the MQWs, a 23.5 nm-thick Zn_0.97_Mg_0.03_O buffer layer and a 39 nm-thick Zn_0.8_Mg_0.2_O barrier were grown on the *c*-plane sapphire substrate. Then, alternating ZnO wells and Zn_0.8_Mg_0.2_O barriers were deposited.

### Identification of structural and optical properties

XRD measurements were performed with a nine-circle diffractometer with an incident wavelength of 1.0331 Å at the IU22 undulator beamline TPS-09A of the Taiwan Photon Source at the National Synchrotron Radiation Research Center. Two pairs of slits between the sample and the LaCl_3_ scintillation detector yield a resolution of over 3.2 × 10^−3^ Å^−1^. A cross-sectional TEM sample with a thickness of approximately 90 ± 10 nm was prepared using a focused ion beam. In addition, the microstructure and interface qualities of the samples were analyzed using scanning transmission electron microscopy (STEM) performed with a JEOL 2100F microscope equipped with an energy-dispersive X-ray detector operating at 200 keV. RT photoluminescence (PL) was measured under the excitation of a He–Cd laser with a wavelength of 325 nm using a monochromator (iHR 550, Horiba Inc.). The transmittance and reflectance spectra were also measured using a white light source (Ocean Optics DH-2000-BAL) and a spectrometer (iHR 320, Horiba Inc.).

### Degenerate pump–probe measurement

**Fig. 1:**
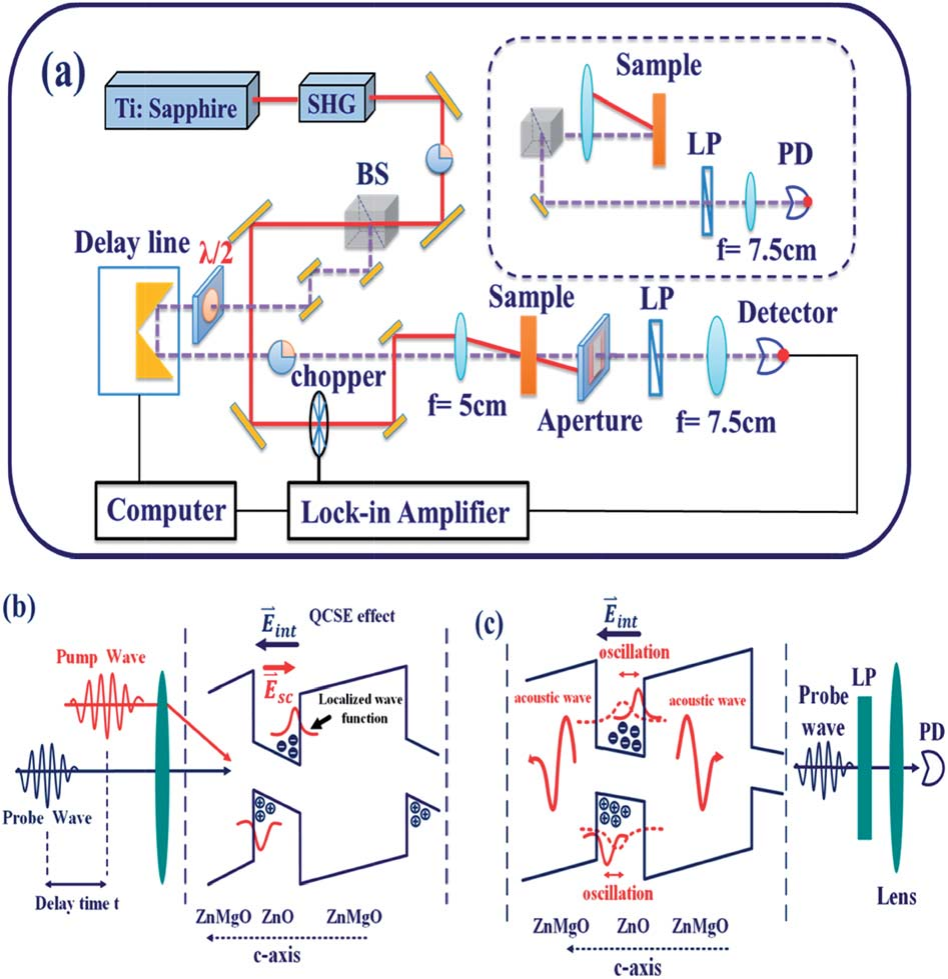
Schematic diagram of (a) the experimental setup for the TDT and TDR degenerate pump–probe measurements and the (b) generation and (c) detection of acoustic waves in ZnO/ZnMgO MQWs.

Fig. 1a illustrates the experimental setup for TDT and TDR (inset) measurements using the degenerate pump–probe technique. A mode-locked Ti:sapphire laser (Tsunami, Spectral Physics Inc.) equipped with a frequency doubler (Model 3980, Spectral Physics Inc.) producing linearly polarized UV pulsed light with 80 MHz repetition rate and 105 fs pulse width was used.

By passing the beam through a beam splitter and a half-wave (*λ*/2) plate, two orthogonal polarized beams were used as the pump beam (red solid line) and the probe beam (purple dashed line), respectively. After passing through a convergent lens, these two beams were focused simultaneously onto the same location on the sample at an angle of 12°. The horizontal polarized pump beam has an average power which has been set from 4 mW to 16 mW or 4 to 16 μJ cm^−2^ and the vertical polarized probe beam has a fixed power of approximately 0.5 mW or 0.5 μJ cm^−2^. The relative time delay between the pump and probe beams was controlled with a high-resolution translation stage. To filter out the residual pump beam, an aperture and a linear polarizer were placed in front of the detector. For the reflection scheme (upper right in Fig. 1a), the probe beam reflected from the sample was divided and collimated by a beam splitter and a mirror. Finally, the transmitted and reflected probe beams were collected by the detector. In order to efficiently increase the signal-to-noise ratio of the received signal, we adopted a lock-in amplifier incorporating a mechanical chopper in the path of the pump beam.

The CAP generation and detection mechanisms from the ZnO/ZnMgO MQWs are illustrated in Fig. 1b and c, respectively. ZnO consists of alternating layers of Zn cations and O anions along the *c*-axis. This, along with the piezoelectric field induced by the strain at the quantum well interfaces due to lattice mismatch between the ZnO and ZnMgO layers, induces a net spontaneous dipole and piezoelectric fields along the *c*-axis. The net internal electric field, *E*_int_, causes band tilting, reducing the exciton oscillator strength through separation of the electron and hole wave functions, and increasing the exciton lifetime. These are referred to as the quantum-confined Stark effects (QCSEs).^20^ After the absorption of the femtosecond UV pump–pulse by the *c*-axis ZnO wells in the MQWs, the periodic distributions of photo-excited carriers, with the electrons and holes localized near the conduction and valence bands, respectively, produce a screening field *E*_sc_ (red arrow in Fig. 1c). Then the internal electric field is perturbed by *E*_sc_ to generate the displacive CAP oscillation,^11^ which propagates in the +*c* and −*c* directions. Therefore, the reflected and transmitted probe beams are modulated by the CAP oscillation, and the oscillation is observable in both the TDR and TDT traces.

## Results and discussion

**Fig. 2:**
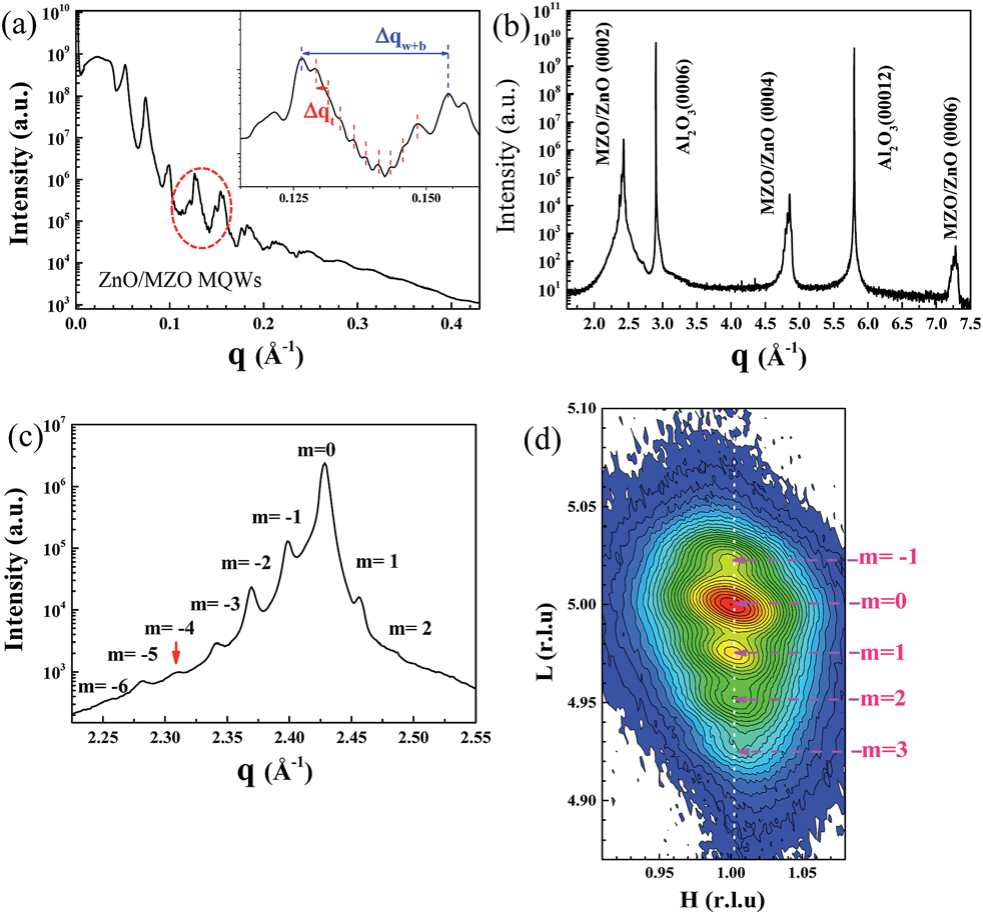
(a) Reflectivity curve of the ZnO/ZnMgO MQWs deposited on a (0001) sapphire substrate. The inset shows the enlarged reflectivity curve of the circled region. (b) XRD radial scan along the surface normal of the ZnO/ZnMgO MQWs grown on a (0001) sapphire substrate. (c) Enlarged XRD radial scan around the ZnO/ZnMgO MQW (0002) diffraction peak. (d) XRD RSM at the (101̄5) ZnO/ZnMgO MQW reflection.

Fig. 2a shows the X-ray reflectivity of the ZnO/ZnMgO MQWs on (0001) sapphire substrates. The reflectivity curve exhibits a typical shape with Bragg peaks separated by Kisseig fringes. The Bragg peaks are correlated with a MQW period of ∼22.76 nm, determined from Δ*q*_w+b_ = 0.276 nm^−1^ (inset of Fig. 2a). The fringes are qualitatively ascribed to the interference between X-rays scattered by the difference in electron density between the well and barrier layers at the interface regime, which is evidence for the vertical periodicity of the composition modulation. Furthermore, the appearance of satellite peaks among the regime of Bragg peaks is ascribed to the difference in electron density between the substrate and the film; the period of the fringes reflects the films’ partial thickness of ∼182 nm, calculated from Δ*q*_t_ = 0.03445 nm^−1^. This result represents the eight-period thickness of the MQWs because the total thickness of the films is beyond the thickness detection limit of X-ray reflectivity. The presence of these clear oscillations indicates that the surface and the interface are well correlated, and smooth enough to produce these oscillations.

A typical radial scan along the surface normal of the ZnO/ZnMgO MQW sample is also illustrated in Fig. 2b. The observation of only MQW (0002), (0004) and (0006) reflections, together with the Al_2_O_3_ (0006) and (00012) reflections, demonstrates that the ZnO/ZnMgO MQW structure is *c*-plane oriented with its [0001] axis parallel to the Al_2_O_3_ [0001] direction. Furthermore, it is worth mentioning that the appearance of well-resolved satellite peaks of the 5^th^ order around the (0002) peak (Fig. 2c) arises from interference between the X-ray waves reflected from the sample structure. This is an indication of the high crystalline quality of the MQW structure, because both interface imperfection and compositional inhomogeneity would decrease the phase coherence and suppress the intensity of the satellite peaks. However, the 4^th^-order satellite peak is missing in the XRD pattern. From a scattering perspective,^23^ the MQW structure can be considered as a one-dimensional artificial crystal that is comprised of a number of unit cells with a period *Λ*_MQW_. Each unit cell consists of two sublayers, *i.e.*, a barrier and a well, with thicknesses *Λ*_barrier_ and *Λ*_well_ and electron densities *ρ*_barrier_ and *ρ*_well_, respectively. It is worth mentioning that certain reflections may be suppressed in the diffraction patterns as a result of destructive interference between the sublayers when the thickness ratio *Λ*_MQW_/*Λ*_well_ is a rational number *m*/*n*, where *m* and *n* are integers. Therefore, the absence of the 4^th^-order satellite peak indicates that the MQW period is approximately four times the thickness of the well layer. The corresponding MQW period (*Λ*_MQW_) derived from the satellite peak spacing, Δ*q* = 0.2805 nm^−1^, is ∼22.4 nm. The thicknesses *Λ*_well_ and *Λ*_barrier_ were estimated to be ∼5.4 and ∼17.4 nm, respectively.

In order to realize peak broadening and determine the overall strain state of the Mg_0.03_Zn_0.97_O buffer layer and the ZnO/ZnMgO MQWs, reciprocal space mapping (RSM) near the asymmetric Zn_0.97_Mg_0.03_O (101̄5) reflection was performed and is shown in Fig. 2d. The unidentifiable (101̄5) reflections between Zn_0.97_Mg_0.03_O and the MQWs in the H–L plane of the RSM indicate that the MQW structure is nearly fully strained; ZnO/ZnMgO MQWs were grown coherently on Zn_0.97_Mg_0.03_O buffer layers. Considering Fig. 2c and d, the average lattice constants, *a* and *c*, of the MQWs can be estimated to be approximately 0.3269 and 0.5174 nm, respectively. In comparison to bulk ZnO, with *a* = 0.3244 and *c* = 0.5204 nm, the lattice of the MQWs experienced tensile strain in the lateral direction of ∼0.77%, but was compressed along the growth direction by approximately −0.58%.

**Fig. 3:**
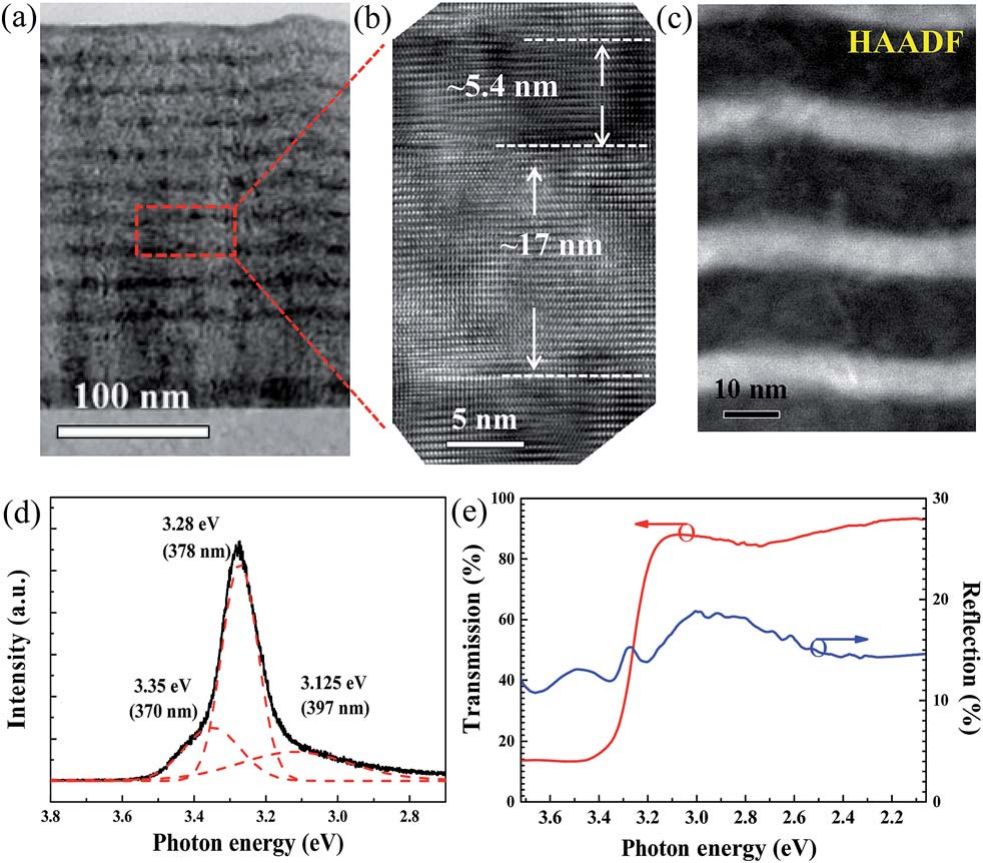
(a) Cross-sectional TEM micrograph recorded along the [101̄0]_Al_2_O_3__ projection. (b) High-resolution image of the ZnO/ZnMgO MQWs in the boxed region shown in (a). (c) Cross-sectional HAADF-STEM image. (d) PL spectra of the ten-pair ZnO/ZnMgO MQW film on (0001) sapphire measured at 300 K. (e) RT transmittance (red solid curve) and reflectance (blue solid curve) spectra of the ten-pair ZnO/ZnMgO MQW film on (0001) sapphire.

To confirm the well width and interface structure between Zn_0.8_Mg_0.2_O and ZnO in the MQWs, a cross-sectional TEM measurement was performed. Fig. 3a and b show low and high magnification TEM micrographs, respectively, along the [101̄0]_Al_2_O_3__ projection. The TEM image also reveals good periodicity and a uniform distribution across the entire 10-pair MQW structure. The estimated period of the MQWs, consistent with the XRD results, is approximately 22.4 nm. Moreover, the estimated thicknesses of the ZnO well and Zn_0.8_Mg_0.2_O barrier layer from Fig. 3b are 5.4 and 17 nm, respectively. These results again illustrate that the 4^th^ satellite peak in the XRD measurement (Fig. 2c) is suppressed because the ratio of *Λ*_MQW_ to *Λ*_well_ is approximately 4. Fig. 3c shows the high-angle annular dark field (HAADF) STEM image, which is known to be highly sensitive to variation in atomic species. The contrast between each ZnO well layer is clearly enhanced, owing to the atomic-number dependence of the HAADF-STEM intensity, and homogeneous epitaxial growth is thereby observed together with the thickness of each ZnO layer. Furthermore, the buckled or wrinkled structure in the HAADF-STEM image is attributed to the larger lattice strain caused by unrelaxed growth of the Zn_0.97_Mg_0.03_O buffer layers.

The RT PL spectrum, as shown in Fig. 3d, was examined to characterize the optical performance of the MQWs. After fitting to a Gaussian profile, the near-band-edge emission of the MQWs can be clearly decomposed into three emission peaks, located at 397 nm, 378 nm and 370 nm. The emission peaks at 378 nm (3.28 eV) and 370 nm (3.35 eV) are dominated by the free exciton (FX) emission of ZnO and Zn_0.97_Mg_0.03_O, respectively. The other strong emission peak at 397 nm, representing surface-bound-exciton emission, originates from the radiative recombination of excitons bound to interface defects in the ZnO layers.^24^ The photoluminescence of the barrier layer was not observed in the PL spectrum because the bandgap of Zn_0.8_Mg_0.2_O is larger than that of the He–Cd pumping source (3.815 eV). To further determine the excitonic recombination energy levels, the transmittance (red solid curve) and reflectance (blue solid curve) spectra were explored, as illustrated in Fig. 3e. The peak at 3.28 eV (corresponding to 378 nm) in the reflectance spectrum, consistent with the PL measurement results, is assigned to the FX of the ZnO well. Thus, considering the exciton binding energy of ∼60 meV, the bandgap of the ZnO layer is ∼3.34 eV (corresponding to 371 nm).

**Fig. 4:**
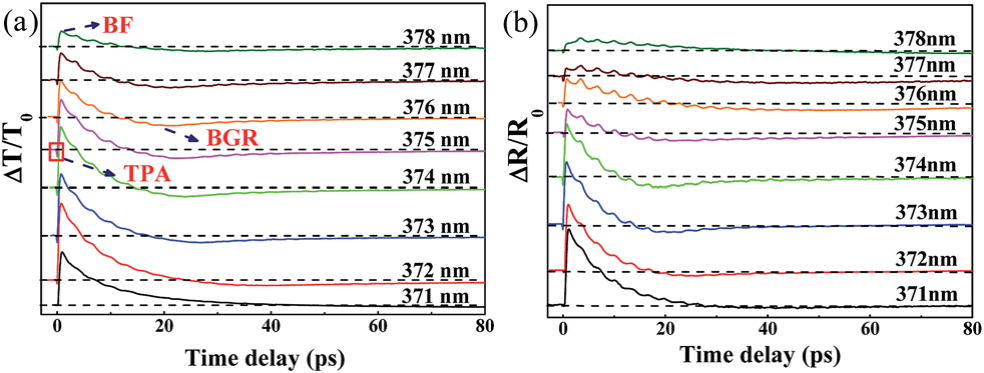
(a) The TDT traces (Δ*T*/*T*) and (b) the TDR traces (Δ*R*/*R*) of ZnO/ZnMgO MQWs for pump wavelengths from 371 nm to 378 nm.

To investigate the behavior of CAP oscillations in ZnO/ZnMgO MQWs around the exciton resonance, TDT and TDR measurements were performed using the degenerate pump–probe technique. Fig. 4a and b show the measured TDT (Δ*T*/*T*) and TDR (Δ*R*/*R*) traces, respectively. Measurements were taken with the excited photon energy of the pump pulse tuned above the exciton resonance (3.28 eV, pump wavelength *λ*_p_ = 378 nm), but below the bandgap of the ZnO layer (3.34 eV, *λ*_p_ = 371 nm). Except for *λ*_p_ at 371 nm, the TDT and TDR signals generally start with a sharp dip around the zero time delay, owing to the two-photon absorption (TPA). The observation of TPA at a below-bandgap excitation of the ZnO well can be attributed to the absorption of a pump photon to create an exciton, followed by ionization and successful absorption of a probe photon.^16^

Additionally, free electron–hole pairs can also be excited by cascade absorption of two pump photons *via* the exciton level. After a dip around the zero time delay, an instantaneous rise is observed in the TDT and TDR traces due to the occupation of the exciton level inhibiting the subsequent absorption of probe photons. This is termed the band-filling (BF) effect. Through carrier–phonon scattering thermalization, the excited free carriers gradually relaxed to low energy levels. Accumulated free carriers at the bottom of the band edges screened the Coulomb potential energy and resulted in shrinkage of the bandgap, termed the bandgap renormalization (BGR). This then caused the increase in absorption of probe photons and led to negative transient transmittance signals. On the other hand, as the pump photon energy was set near the band edge of ZnO, *i.e.*, *λ*_p_ = 371 nm, the photo-excited carriers occupied the band-edge states and prevented further absorption of probe photons. Therefore, only positive BF was detected in the TDT and TDR signals. In addition to the aforementioned ultrafast carrier dynamics of MQWs, obvious oscillations were superimposed on both the TDT and TDR traces. The oscillations arose from light scattering through the periodic distribution of the photo-excited carrier population induced by the *c*-axis MQW structure with the wave vector *q* = 2π/*Λ*_MQW_ which enabled the observation of coherent oscillations of zone-folded acoustic phonons.

**Fig. 5:**
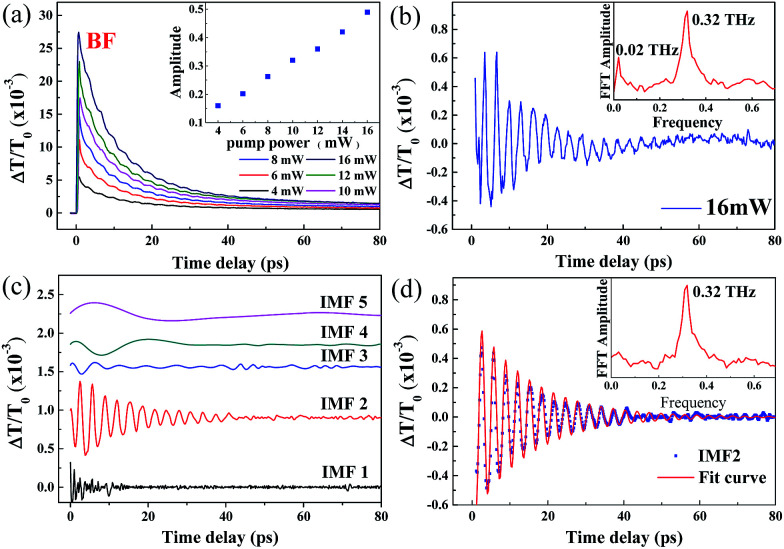
(a) Pump-power-dependent TDT traces and CAP oscillation amplitude (inset) for a pump wavelength of 371 nm. (b) The CAP oscillation extracted from the TDT trace with *P*_p_ at 16 mW and the corresponding FFT spectrum (inset). (c) The extracted intrinsic mode functions (IMF1 to IMF5) for the CAP oscillation and (d) IMF2 from the CAP oscillation and the fitting curve described by eqn (2) and the FFT spectrum (inset).

The evolution of the TDT traces at *λ*_p_ = 371 nm with increasing pump power (*P*_p_) (from 4 mW to 16 mW, or 4 to 16 μJ cm^−2^) at a fixed probe power (approximately 0.5 mW, or 0.5 μJ cm^−2^) is shown in Fig. 5a. It is worth noting that no negative signal resulting from the BGR effect was observed in these traces. All the TDT traces exhibited a positive peak resulting from the BF effect, followed by a gradual decay that can be fitted well using the bi-exponential formula:^15^1
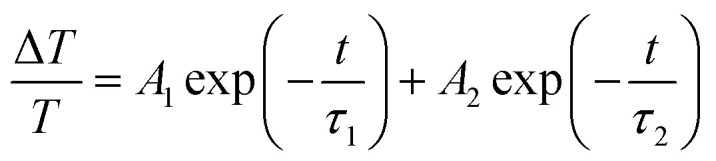
where *A*_1_ and *A*_2_ are scaling factors and *τ*_1_ and *τ*_2_ represent fast and slow decay time constants of approximately 6 ps and 20 ps, respectively. The time constant *τ*_1_ is responsible for the relaxation of free electrons to the bottom of the conduction band *via* electron–phonon scattering and *τ*_2_ is attributed to the non-radiative carrier recombination time.^25^ After subtraction of the fitted curve from the measured TDT trace at *P*_p_ = 16 mW, we obtained a damped oscillation signal with an amplitude of approximately 10^−4^ (Fig. 5b), which is attributed to the CAPs. After applying a fast Fourier transformation (FFT), the spectrum (inset of Fig. 5b) indicates two distinctive oscillation frequencies at approximately 0.02 THz and 0.32 THz. However, the damped oscillation was superimposed with noise and modulated by a low-frequency signal from the environment. In order to filter out these ambiguous effects we employed the empirical mode decomposition (EMD) method proposed by Huang *et al.*^21,22^ to analyze our signals. The decomposition is based on the assumption that each data set consists of different simple intrinsic mode functions (IMFs), the definition of which requires that (i) the number of extrema and zero-crossings in the whole data set must either be equal or differ at most by one and (ii) the mean values of the envelope defined by the local maxima and the envelope defined by the local minima are zero. Therefore, any complicated data set can be decomposed into a finite and often small number of IMFs that admit well-behaved Hilbert transforms. This provides a new method for analyzing nonlinear and non-stationary data.

The decomposed IMFs (IMF1 to IMF5) from the damped oscillation signals of Fig. 5b are shown in Fig. 5c. The first IMF (IMF1) obviously results from the noise. The higher-order IMFs with low amplitude, such as IMF3 to IMF4, might contribute, but only slightly, to the CAP oscillation. IMF5, with a low oscillation frequency of approximately 0.02 THz, may result from the echo of the acoustic wave in the MQW configuration. Thus, the CAP oscillation mainly results from IMF2 (the blue dots in Fig. 5d), which can be fitted well using the damped oscillation formula:2
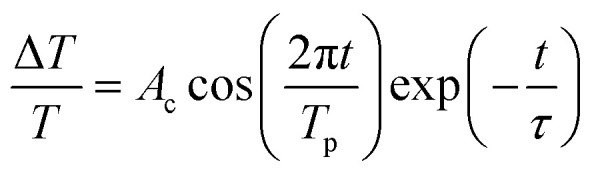
where *A*_c_ = 0.5, *T*_p_ = 0.32 ps and *τ* = 17 ps are the amplitude, period and decay constant of the CAP oscillation, respectively. In addition, the oscillation amplitude *A*_c_ exhibits a linear increase with pump power after fitting, as shown in the inset of Fig. 5a. As seen previously in superlattices,^6^ the period of each pair inside the MQW structure (*Λ*_MQW_) is given by the formula *Λ*_MQW_ = *c*_SL_ × *T*_p_. As the velocity *c*_SL_ of longitudinal acoustic waves inside ZnO^26^ is approximately 6400 m s^−1^ and the oscillation period *T*_p_ is 0.32 ps, the estimated period of the MQWs is approximately 21 nm, close to the value of 22.4 nm measured with XRD and TEM. Thus, the oscillation from the ZnO/ZnMgO MQWs in our pump–probe measurement can be attributed to the zone-folded acoustic phonon mode.

**Fig. 6:**
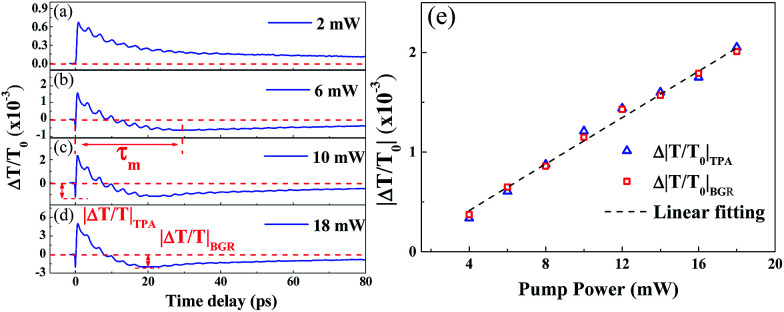
(a–d) TDT traces for pump power increasing from 2 mW to 18 mW for a pump wavelength of 375 nm. (e) Modulation depths of TPA (|Δ*T*/*T*_0_|_TPA_) and BGR (|Δ*T*/*T*_0_|_BGR_) as a function of pump power.

The TDT traces of ZnO/ZnMgO MQWs at various pump powers (*P*_p_ = 2 to 18 mW) with the excited photon energy set near the exciton resonance of the ZnO well layer at 3.31 eV (*λ*_p_ = 375 nm) are shown in Fig. 6a–d. At a lower pump power (*P*_p_ = 2 mW), the TDT trace in Fig. 6a reveals only a positive peak caused by the BF effect, and then gradually relaxes due to carrier–phonon scattering. As the pump power is increased above 4 mW, the TPA dip and the negative |Δ*T*/*T*_0_|_BGR_ value due to the BGR effect can both be observed in the TDT traces (Fig. 6b–d). As we further increase the pump power, the maximum modulation depths of the TDT traces, such as |Δ*T*/*T*_0_|_TPA_ (labeled in Fig. 6c), caused by the TPA at the zero time delay and |Δ*T*/*T*_0_|_BGR_ (labeled in Fig. 6d), caused by the BGR at a certain time delay (*τ*_m_, the BGR buildup time), become obvious. Moreover, *τ*_m_ decreases as the pump power increases.

The TPA effect around the exciton resonance can be reasonably explained by the cascaded absorption of two pump photons or the simultaneous absorption of one pump and one probe pulse around the zero time delay, exciting the electron from the valence band (VB) to the exciton level and then to the conduction band (CB).^15,16^ This is termed the resonance TPA. Unlike non-resonance TPA, caused by the virtual transition and exhibiting a nonlinear relation with *P*_p_, the modulation depths of TPA (|Δ*T*/*T*_0_|_TPA_) around the zero time delay and BGR (|Δ*T*/*T*_0_|_BGR_) at *τ*_m_ increase linearly with the pump power and are consistent with each other, as shown in Fig. 6e. For BGR, most free electrons can be excited *via* the exciton level to the CB, leaving holes in the VB, by means of the cascaded absorption of two pump photons. Through carrier–phonon scattering, the cooled carriers occupy the lower lying electronic states at the bottom of the CB and/or the hole states at the top of the VB, as has been previously reported in polar ZnO.^16^ Then, the Coulomb potential energy is screened, resulting in the BGR effect. This is the reason why the modulation depth |Δ*T*/*T*_0_|_BGR_ is proportional to |Δ*T*/*T*_0_|_TPA_. As pump power increases, more free electrons are excited to the CB at an earlier time, speeding up electron–electron and electron–phonon scattering. Similar processes also occur for the excited holes in the VB. Thus, the free carriers relax quickly to the band edge and cause the BGR to occur early at the higher pumping powers.

**Fig. 7:**
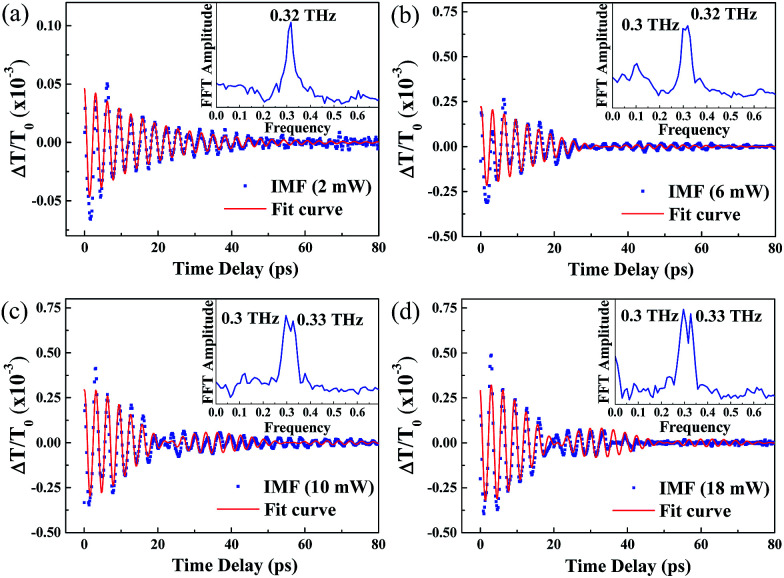
The CAP oscillation extracted from the TDT trace for a pump wavelength of 375 nm and pump power of (a) 2 mW, (b) 6 mW, (c) 10 mW and (d) 18 mW.

Fig. 7a–d show the retrieved IMFs of CAP oscillations from the TDT traces in Fig. 6a–d. The blue dots in Fig. 7a reveal a monotonically damped IMF CAP oscillation at *P*_p_ = 2 mW. The IMF mode can be fitted well using eqn (2) (red line) with a single oscillation frequency of approximately 0.32 THz, as shown in the FFT spectrum (inset of Fig. 7a). The IMFs retrieved when the pump power was increased from 6 mW to 18 mW are shown by the blue dots in Fig. 7b–d. In contrast to Fig. 7a, the beating of two distinct oscillation signals is shown in these plots. Thus, we fitted the damped traces with a formula containing a superposition of two oscillation signals:3
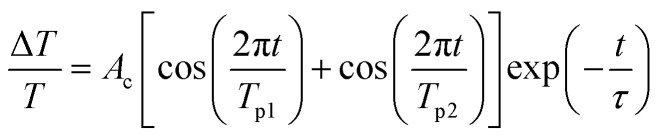
where *A*_c_ is a scaling factor, *T*_p1_ and *T*_p2_ are the periods of the two oscillation signals and *τ* is a decay constant. The well-fitted curves are shown in red in Fig. 7b–d. The corresponding FFT spectra (the insets) reveal that the amplitude of the additional oscillation, with a frequency of approximately 0.3 THz, increased with pump power. The occurrence of the additional oscillation frequency should correlate with the BGR which produces the negative TDT traces, as shown in Fig. 6b–d. Theoretically, the induced refractive index change (Δ*n*) from the BGR is negative (Δ*n* < 0), opposite to the BF effect with Δ*n* > 0. Therefore, based on Thomsen’s model,^27^ the oscillation frequency of CAP can be expressed by the formula *ν* = (2*nc*_SL_)/*λ*_p_. When Δ*n* < 0, *n* decreases to cause a reduction in the oscillation frequency. In addition, the amplitude of the low frequency peak at 0.3 THz is slightly larger than that of the peak at 0.32 THz because of the longer dwell time in the BGR state than in the BF state, as shown in Fig. 6c and d.

**Fig. 8:**
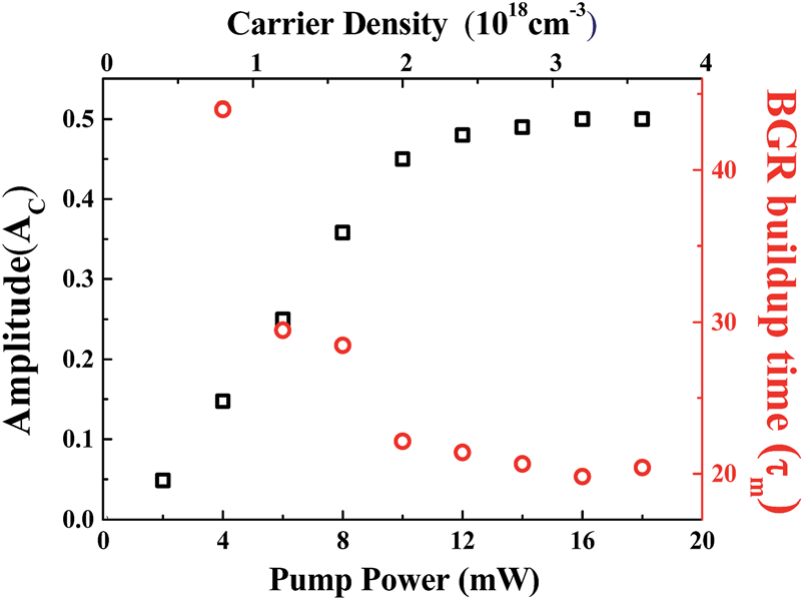
The amplitude of CAP oscillations *A*_c_ and BGR buildup time (*τ*_m_) as a function of pump power.

Fig. 8 shows the amplitude of CAP oscillation as a function of pump power or excited carrier density (*N*_exc_). Here, the excited carrier density is estimated using the formula *N*_exc_ = *I*_exc_*α*/*ℏω*_exc_,^22,25^ where *I*_exc_ is the pumping fluence (or energy flux density), *α* is the absorption coefficient and *ℏω*_exc_ is the photon energy. The absorption coefficient *α* can be obtained from the transmittance *T*_0_ (Fig. 3e) by the formula:^28^4
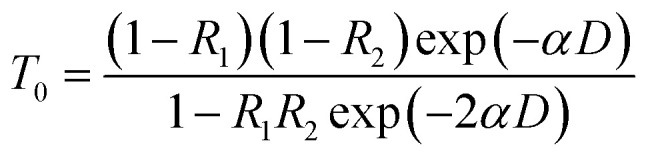
where *R*_1_ is the reflectance between air and the ZnO, *R*_2_ is the reflectance between the ZnO and the sapphire and *D* is the thickness of the well. From Fig. 3e, the obtained value for *α* is approximately 1.12 × 10^5^ cm^−1^ at a pump wavelength of 375 nm. The diameters of the focused pump and probe beams were measured to be 42–45 μm. In contrast to the linear dependence of the modulation depth of TPA and BGR on the pump power (Fig. 6e), the amplitude *A*_c_ of CAP oscillation (Fig. 8) increases almost linearly with pump power *P*_p_ at lower excited carrier densities, but saturates as *P*_p_ increases beyond 10 mW.

By analyzing the initial phase of CAP oscillation near the exciton resonance and bandgap, as shown in Fig. 5 and 7, the cos(*ω*_phonon_*t*) dependence was observed. Based on the mechanism of coherent phonon generation^29,30^ the CAP oscillations of the ZnO well layers in the polar ZnO/ZnMgO MQWs are induced *via* DECP. Theoretically, the driving force of a harmonic oscillator can be expressed by the sum of the Raman term and the nonlinear longitudinal polarization.^29,30^ The nonlinear polarization can be attributed to the second- and the third-order nonlinear susceptibilities as well as a longitudinal polarization generated by ultrafast drift-diffusion currents, which can excite coherent phonons *via* transient depletion field screening (TDFS), and the polarization associated with coherent electronic wavefunctions.^29,30^

Generally, more carriers are excited at higher pump powers that perturb the built-in electric field, enhancing the amplitude of CAP. Nevertheless, the built-in electric field should be screened by the photo-induced carriers to decrease ultrafast drift-diffusion currents *via* TDFS,^31^*i.e.*, reducing the band-tilted effect. Therefore, when the screening of the built-in electric field by photon-induced carriers is carried out at a higher excited carrier density for polar ZnO/ZnMgO MQWs, it will cause the decline of nonlinear polarization and reduce the driving force of harmonic oscillation. Hence the saturation of the CAP amplitude is observable. Fig. 8 also shows the BGR build-up time *τ*_m_, the relative time delay at the TDT trace minimum, as a function of excited carrier density. It reveals that *τ*_m_ decreases linearly with increasing carrier density and levels off as the carrier density increases beyond 2 × 10^18^ cm^−3^ (or *P*_p_ = 10 mW) through the cascade TPA absorption, *i.e.*, the internal electric field would be fully screened when the number of excited carriers is beyond 2 × 10^18^ cm^−3^. Like the aforementioned results in Fig. 1b and c, the periodic distribution of excited carriers from the TPA produces electronic strain that couples with the selective zone-folded acoustic-phonon modes *via* DECP. With further increases in the pump power, more free carriers are excited and the amplitude of CAP oscillation is enhanced. On the other hand, the higher carrier density causes fast cooling of the high-lying carriers to the band edge *via* carrier–carrier scattering and carrier–phonon scattering, leading to the fast build-up of BGR. At a relatively high carrier density the built-in field might also be screened, flattening the potential well of the MQW structure and suppressing the increase in *A*_c_. Therefore, the flattening of the potential well, rather than the carrier density, is responsible for the saturation of *A*_c_ and *τ*_m_. The carrier density accounts for the linear dependence of BGR modulation depth on the pump power in Fig. 6e.

## Conclusions

In this work, we grew ZnO/Zn_0.8_Mg_0.2_O multiple quantum wells (MQWs) with high structural quality and regularly arranged well and barrier layers using pulsed laser deposition, as evidenced by high-resolution cross-sectional transmission electron microscopy (TEM) images and the pronounced high-order satellite peaks in X-ray crystal truncation rods. The thicknesses of the well and barrier layers can be estimated from the satellite peak spacing and the suppressed intensity of the 4^th^-order satellite peak in the X-ray diffraction (XRD) pattern. They are approximately 5.4 and 17.4 nm, respectively, and are consistent with the TEM image. When the excited photon energy was set between the band edge and the exciton resonance, sub-THz coherent acoustic phonon (CAP) oscillation from the ZnO/Mg_0.2_Zn_0.8_O MQWs was demonstrated in a degenerate pump–probe measurement. The occurrence of acoustic waves is mainly attributed to the perturbation of the internal electric field resulting from photo-excited carriers based on cascade two-photon absorption. By applying empirical mode decomposition, the purified signal can be retrieved to calculate the CAP oscillation frequency of ∼0.32 THz and the MQW period of ∼21 nm, consistent with the XRD and TEM results. With the increase in photo-excited power (or excited carriers), an additional oscillation frequency of the acoustic wave, 0.3 THz, was observed because the bandgap renormalization effect caused a negative change in refractive index (Δ*n* < 0) in the MQWs. Furthermore, the saturation of CAP amplitude at high pumping power is attributed to the screening of the built-in electric field by the generation of many photo-excited carriers.

## Conflicts of interest

There are no conflicts to declare.

## Supplementary Material

## References

[cit1] Daly B. C., Holme N. C. R., Buma T., Branciard C., Norris T. B., Tennant D. M., Taylor J. A., Bower J. E., Pau S. (2004). Appl. Phys. Lett..

[cit2] Lin K. H., Yu C. T., Sun S. Z., Chen H. P., Pan C. C., Chyi J. I., Huang S. W., Li P. C., Sun C. K. (2006). Appl. Phys. Lett..

[cit3] Zeiger H. J., Vidal J., Cheng T. K., Ippen E. P., Dresselhaus G., Dresselhaus M. S. (1992). Phys. Rev. B: Condens. Matter Mater. Phys..

[cit4] Merlin R. (1997). Solid State Commun..

[cit5] Garret G. A., Albrecht T. F., Whitaker J. F., Merlin R. (1996). Phys. Rev. Lett..

[cit6] Ruello P., Gusev V. E. (2015). Ultrasonics.

[cit7] Kini R. N., Kent A. J., Stanton N. M., Henini M. (2006). Appl. Phys. Lett..

[cit8] Wright O. B., Perrin B., Matsuda O., Gusev V. E. (2001). Phys. Rev. B.

[cit9] Babilotte P., Ruello P., Mounier D., Pezeril T., Vaudel G., Edely M., Breteau J.-M., Gusev V., Blary K. (2010). Phys. Rev. B: Condens. Matter Mater. Phys..

[cit10] Young E. S. K., Akimov A. V., Campion R. P., Kent A. J., Gusev V. (2013). Phys. Rev. B: Condens. Matter Mater. Phys..

[cit11] Sun C. K., Liang J. C., Yu X. Y. (2000). Phys. Rev. Lett..

[cit12] Sanders G. D., Stanton C. J., Kim C. S. (2001). Phys. Rev. B: Condens. Matter Mater. Phys..

[cit13] Lim J. H., Kang C. K., Kim K. K., Park I. K., Hwang D. K., Park S. J. (2006). Adv. Mater..

[cit14] Lu T. C., Lai Y. Y., Lan Y. P., Huang S. W., Chen J. R., Wu Y. C., Hsieh W. F., Deng H. (2012). Opt. Express.

[cit15] Lin J. H., Su H. J., Lu C. H., Chang C. P., Liu W. R., Hsieh W. F. (2015). Appl. Phys. Lett..

[cit16] Lin J. H., Liu W. R., Lin Y. C., Su H. J., Chen H. R., Tsai C. Y., Chen Y. H., Hsieh W. F. (2016). AIP Adv..

[cit17] Sun C. K., Sun S. Z., Lin K. H., Zhang K. Y. J., Liu H. L., Liu S. C., Wu J. J. (2005). Appl. Phys. Lett..

[cit18] Ou P. C., Lin J. H., Chang C. A., Liu W. R., Hsieh W. F. (2010). J. Phys. D: Appl. Phys..

[cit19] Lin J. H., Shen Y. K., Liu W. R., Lu C. H., Chen Y. H., Chang C. P., Lee W. C., Hong M., Kwo J. R., Hsu C. H., Hsieh W. F. (2016). J. Phys. D: Appl. Phys..

[cit20] Davis J. A., Jagadish C. (2009). Laser Photonics Rev..

[cit21] Huang N. E., Shen Z., Long S. R., Wu M. C., Shih H. H., Zheng Q., Yen N., Tung C. C., Liu H. H. (1998). Proc. R. Soc. London, Ser. A.

[cit22] Huang N. E., Long S. R., Shen Z. (1996). Adv. Appl. Mech..

[cit23] Willmott P. , An Introduction to Synchrotron: Techniques and Application, Wiley, New York, 2011

[cit24] Kuo C. C., Lin B. H., Yang S., Liu W. R., Hsieh W. F., Hsu C.-H. (2012). Appl. Phys. Lett..

[cit25] Ou P. C., Liu W. R., Ton H. J., Lin J. H., Hsieh W. F. (2011). J. Appl. Phys..

[cit26] Lu Y. , Emanetoglu N. W. and Chen Y., in Zinc Oxide Bulk, Thin Films and Nanostructures, ed. C. Jagadish and S. J. Pearton, Elsevier, New York, 2006, ch. 13, pp. 443–489

[cit27] Thomsen C., Grahn H. T., Maris H. J., Tauc J. (1986). Phys. Rev. B: Condens. Matter Mater. Phys..

[cit28] Ahmad J., Minami H., Alam S., Yu J., Arai Y., Uwe H. (2008). Chin. Phys. Lett..

[cit29] Ishioka K. and Misochko O. V., in Progress in Ultrafast Intense Laser Science, ed. A. Giulietti, and K. Ledingham, Springer-Verlag, Berlin Heidelberg, 2010, vol. V, ch. 2, pp. 23–46

[cit30] Dekorsy T. , Cho G. C., and Kurz H., in Light Scattering in Solids VIII, ed. M. Cardona and G. Güntherodt, Springer-Verlag, Berlin Heidelberg, 2001, vol. 76, ch. 4, pp. 169–209

[cit31] Dekorsy T., Pfeifer T., Kütt W., Kurz H. (1993). Phys. Rev. B: Condens. Matter Mater. Phys..

